# Allostatic load and risk of prostate cancer in UK Biobank

**DOI:** 10.1038/s41598-025-21510-8

**Published:** 2025-10-27

**Authors:** Jie Shen, Yufan Guan, Bernard F. Fuemmeler, Lisa S. Shock, Hua Zhao

**Affiliations:** 1 Department of Public Health Sciences, School of Medicine, University of Virginia, Charlottesville, VA 22903 USA; 2 Departments of Family Medicine, School of Medicine, Virginia Commonwealth University, Richmond, VA 23284 USA; 3 Departments of Microbiology and Immunology, School of Medicine, Virginia Commonwealth University, Richmond, VA 23284 USA

**Keywords:** Cancer, Risk factors

## Abstract

Allostatic load (AL), a biomarker of chronic stress, has been implicated in the carcinogenesis of various types of cancers. However, its role in the etiology of prostate cancer is unclear. Utilizing valuable resources from the UK Biobank, we analyzed the association between AL and subsequent risk of prostate cancer in a total of 161,964 men. Levels of AL were higher in cases than in non-cancer controls (3.47 vs. 3.35, P < 0.001). In the univariate analysis, one unit increase in AL was associated with a 5% increased risk of prostate cancer (hazard ratio (HR) = 1.05, 95% Confidence Interval (CI) 1.03, 1.06). In the multivariate Cox regression analysis, the significant association was only observed among men younger than 58 years old. The significant association was further confirmed in the categorical analysis, and compared to men with AL of 0, those with AL of 1, 2, 3, 4, 5, and ≥ 6 had a statistically significantly increased risk of prostate cancer, respectively. Additionally, we observed a more than additive joint effect between AL and polygenic risk score (PRS) of prostate cancer among younger men (< 58 years old). In summary, those findings have shown that higher AL was associated with an increased prostate cancer risk in younger men.

**Supplementary Information:**

The online version contains supplementary material available at 10.1038/s41598-025-21510-8.

## Introduction

Prostate cancer is one of the most diagnosed cancers among men worldwide. In the United States, it is expected that 299,010 men will be diagnosed with prostate cancer and 35,250 men will die from prostate cancer in 2024^1^. The consensus risk factors for prostate cancer include age, family history of prostate cancer, race/ethnicity, and genetic factors discovered in genome-wide association studies^2^. In addition, obesity, certain dietary factors (e.g., fruits and vegetables, whole grain, high-fat dairy, red meat, and processed foods), and exposure to certain chemicals (e.g., cadmium, herbicides, and pesticides) have been linked to prostate cancer risk. However, the evidence was inconclusive^2^.

Chronic stress has long been linked to cancer development^3^. In mice, repeated stress has been linked to an increased expression of genes related to prostate proto-oncogenes in prostate^4^ and accelerated prostate cancer development^5^. A few prospective cohort studies have examined the association between psychological stress and prostate cancer risk^6,7^. For example, among first responders in New York City, re-experiencing the stressful events of 9/11 was associated with increased prostate cancer incidence^6^. However, in another cohort study from Denmark, psychological stress was not associated with prostate cancer^7^. The difference may be due to the different instruments used to measure psychological stress. Questionnaire-based instruments may not account for the individual’s difference in resilience and the biological response to the stress.

Since introduced by Dr. Bruce McEwen in the 1990s^8,9^, the term “allostatic load” has been extensively studied in its relationship to chronic diseases, including cardiovascular disease, diabetes, cancer, etc.^10–12^. Allostatic load (AL) describes the cumulative physiological toll on the body due to chronic stress^8^. It reflects the wear and tear on various biological systems resulting from prolonged exposure to stressors. AL incorporates multiple physiological systems, including the nervous, endocrine, immune, and cardiovascular systems. Chronic stress can lead to dysregulation in these systems and leads to AL. Because AL quantifies the biological consequence of chronic stress, it offers an alternative to the traditional questionnaire-based instruments to assess chronic stress.

The role of AL in prostate cancer carcinogenesis has not been explored. The only report is from a meeting abstract, which reports from a small cross-sectional study that shows higher AL is associated with more aggressive phenotypes of prostate cancer, including de novo metastatic disease, earlier progression to castrate resistance and higher Gleason scores^13^. To fill the gap,

We performed the first study to investigate the association between AL and incident prostate cancer risk, using the resource from the UK Biobank. We hypothesize that higher AL was associated with an increased prostate cancer risk in men.

## Methods

### Study cohort

During 2006–2010, the UK Biobank followed up over 500,000 volunteers in the UK aged 46–69 years from the general population^14^. At baseline, participants completed a touch-screen questionnaire, provided biological samples, and had physical measurements across the UK. Informed consent has been obtained from all subjects. For our study, we identified 212,513 male participants as the study cohort. We included only men who had no history of cancer diagnosis at enrollment. Men with a history of benign tumors, carcinoma in situ, nonmelanoma skin cancer, and unknown prevalent cancers were excluded. Additionally, we excluded men who had incomplete information on any of the 11 factors used to construct AL scores. Therefore, 161,964 men were left for the final analysis.

### Prostate cancer cases ascertainment

This study identified incident prostate cancer cases using the ICD-10 code reported in the UK Biobank database (Supplement Table 1). Men were followed up until the censoring date (12/31/2020), with a median follow-up time of 11.6 years. Incident prostate cancer cases were defined as men first diagnosed with malignant neoplasms of the prostate during the follow-up. Additionally, we excluded men who were diagnosed with prostate cancer within one year after their initial enrollment (N = 676). In total, 7291 men with incident prostate cancer were identified from the whole study population (N = 161,964).

### AL score construction

In this study, we used eleven factors to construct the AL score from measurements collected from the baseline. The detailed methods were described by Zhao and Chyu et al.^15–17^. Biomarkers for AL were derived from three cardiovascular (Systolic blood pressure (SBP), diastolic blood pressure (DBP), and pulse rate (PR)), one inflammatory (C-reactive protein (CRP)), one kidney (creatinine), and six metabolic markers (high-density lipids (HDL), low-density lipids (LDL), abnormal cholesterol, triglyceride (TG), hemoglobin A1C (HbA1c), and waist to hip ratio (WHR)). Each biomarker was cut off by the clinical threshold and awarded either 0 (low risk) or 1 (high risk). Moreover, we also considered the history of medication for metabolic diseases and hypertension. Anyone with a history of medications for metabolic disease or hypertension interpreted the medication factor as a “yes” and was awarded a 1; otherwise was awarded a 0. Detailed information on those 12 factors is shown in the Supplement Table 2. To illustrate the potential influence of age on each component of the allostatic load score, we examined the association between age and each of the 11 individual biomarkers, stratified by case–control status (Supplementary Table 3). The AL score was accumulated by all selected factors, ranging from 0 to 11. AL was first treated as a continuous variable. In further analysis, we treated AL as a categorical variable.

### Assessment of covariates

Covariates included baseline demographics (age of disease diagnoses (for the cases) or last follow-up (for the controls and race), family history of prostate cancer, socioeconomic status (SES) (education, employment, income, and Townsend deprivation index), lifestyle factors (cigarette smoking, alcohol consumption, sleep quality, and physical activity), and prostate cancer polygenetic risk score (PRS). The Townsend deprivation index was derived from postcodes and classified as low and high by median. Family history of prostate cancer was accessed by whether the brother or father has had a history of prostate cancer. Education was classified as “High school or less” and “College/professional “. Income was classified as “less than £39,999” and “over £39,999”. Men’s smoking status was derived using “Current tobacco smoking” and “Past tobacco smoking.” The alcohol consumption was estimated by alcohol intake frequency, classified as “special occasions or never,” “moderate,” and “heavy.” Men’s leisure physical activity was assessed based on MET scores and categorized into “Low,” “Moderate,” and “High.” Additionally, men’s sleep quality was assessed by the presence or absence of insomnia or insomnia, categorized as “never/rarely, “sometimes,” and “usually.” The PRS for prostate cancer were obtained directly from the UK Biobank. Details of how to generate PRS have been previously described by Thompson et al.^18^. We defined the high-risk group as the top 10% of the PRS distribution, based on the study by Seibert et al.^19^, which demonstrated that men in the top 10% have approximately 2–3 times the risk of prostate cancer compared to those at median PRS levels.

### Statistical analysis

First, we compared differences in the mean (for continuous variables) or distribution (for categorical variables) of each covariate between prostate cancer cases and cancer-free controls. Next, we compared the distribution of AL between prostate cancer cases and cancer-free controls. AL was treated as a continuous. The student’s T-test was used to detect differences between continuous variables; the Chi-square test was used to detect differences between categorical variables. Furthermore, the Student’s T-test was performed to evaluate the difference in AL in each category of the covariates between the cancer-free control and case groups. At the same time, the Student’s T-test or ANOVA was used to detect differences between two or more two categories within each covariate. After that, the Cox Proportional Hazard regression model was used to assess the association between the AL and prostate cancer risk. The event in this study was the first diagnosis of prostate cancer. For prostate cancer cases, follow-up time was defined as from baseline to the date of prostate cancer diagnosis. For those who developed other cancers (regardless of invasive or non-invasive), the follow-up time was defined from the baseline to the date of diagnosis and censored at the time of diagnosis. For those lost during the follow-up, the follow-up time was defined from the baseline to the date of the last follow-up. The proportional hazards assumption was tested. If the assumption were violated, we would use the non-proportional hazards model instead. The Likelihood Ratio Test was used to assess the model fitting. We also used Kaplan–Meier survival curves with the log-rank test to determine prostate cancer risk difference between different AL groups. In the multivariate Cox Proportional Hazard regression model, we adjusted demographics (age and race), family history of prostate cancer, education, Townsend deprivation index, lifestyle factors (cigarette smoking, alcohol consumption, sleep quality, and physical activity), and PRS of prostate cancer. To explore the impact of age on the relationship between AL and prostate cancer, we performed the stratified analysis by the median age (58 years old). Finally, we assessed the joint effect between AL and PRS of prostate cancer among younger men. We treated AL as a categorical variable (0: AL of 0, 1: AL > 0). All statistical tests were two-sided, and p-values of less than 0.05 were considered statistically significant. The examinations were conducted using R, version 4.3.0.

## Results

A total of 161,964 men were included in the analysis, with a median of 11.6 years of follow-up. Within that, there were 7,291 incident prostate cancer cases and 154,673 cancer-free men at the end of 2020. The mean follow-up time for the cases was 7.0 years. The distribution of the selected demographics, family history of prostate cancer, SES, and healthy behaviors between cases and non-cancer controls are shown in Table 1. Compared to the controls, prostate cancer cases were more likely to be older (61.0 vs. 56.2 years old, P < 0.001), White (95.78% vs. 94.64%, P < 0.001), and retired (45.45% vs. 27.39%, P < 0.001), having a family history of prostate cancer (16.79% vs. 10.34%, P < 0.001), above high school education (49.13% vs. 46.37%, P < 0.001), and a lower income (49.54% vs 43.72% < P < 0.001), and living in the area with lower degree of deprivation (54.59% vs 50.01%, P = 0.001). In terms of healthy behaviors, prostate cancer cases were more likely being physically active (82.25% vs. 80.87%, P < 0.001), heavy drinkers (55.87% vs. 51.59%, P < 0.001), and having sleeplessness (71.56% vs. 69.28%, P < 0.001). In addition, PRS for prostate cancer was higher among prostate cancer cases than controls (1.25 vs. 0.53, P < 0.001). No significant difference was observed for cigarette smoking.

**Table 1:** Comparison of selected characteristics between prostate cancer cases and controls.

Variables	Cancer-free controls(N = 154,673)	Incident cases (N = 7291)	P value
	Number (%)	Number (%)	
Age at recruitment, mean (SD)	56.2 (8.22)	61.0 (5.83)	< 0.001
Years from recruitment to cancer diagnosis, mean (IQR)		7.0 (4.2, 9.4)	
Race/ethnicity (%)			< 0.001
White	145,794 (94.64)	6,948 (95.78)	
Black	1,973 (1.28)	149 (2.05)	
Asian	4,180 (2.71)	88 (1.21)	
Mixed or others	2,094 (1.36)	69 (0.95)	
Family history of cancer (%)			< 0.001
Yes	9,742 (10.34)	747 (16.79)	
No	84,467 (89.66)	3,701 (83.21)	
Education (%)			< 0.001
High school or less	68,439 (53.63)	2,963 (50.87)	
College/professional	59,182 (46.37)	2,861 (49.13)	
Employment status (%)			< 0.001
Unemployed	11,303 (6.88)	337 (4.66)	
Employed	96,950 (59.03)	3,611 (49.89)	
Retired	44,982 (27.39)	3,290 (45.45)	
Income (%)			< 0.001
Less than £30,999	60,467 (43.72)	3,215 (49.54)	
Over £31,000 to £51,999	77,846 (56.28)	3,275 (50.46)	
Townsend deprivation Score (%)			0.001
Low	77,258 (50.01)	3,976 (54.59)	
High	77,212 (49.99)	3,308 (45.41)	
Cigarette smoking (%)			0.287
Never	75,880 (49.23)	3,526 (48.59)	
Ever	78,243 (50.77)	3,730 (51.41)	
Physical activity (%)			0.007
Low	25,064 (19.13)	1,102 (17.75)	
Moderate/high	105,915 (80.87)	5,105 (82.25)	
Alcohol consumption (%)			< 0.001
Never, light, or moderate	74,801 (48.41)	3,217 (44.13)	
Heavy	79,727 (51.59)	4,072 (55.87)	
Sleeplessness (%)			< 0.001
Never/rarely	47,440 (30.72)	2,072 (28.44)	
Sometimes/frequent	107,005 (69.28)	5,213 (71.56)	
PRS, mean (SD)	0.53 (1.00)	1.25 (0.98)	< 0.001

The distribution of AL scores in the prostate cancer cases and controls is presented in Table 2. The range of AL was from 0 to 11. Overall, the distribution of AL presented a reversed U-shaped trend from 0 to 11, with the peak number et al. of 3, including 22.80% of the cases and 22.22% of the controls. Approximately 2.21% of prostate cancer cases and 3.70% of controls had an allostatic load score of zero, indicating no elevated risk factors. Meanwhile, 10.23% of the cases and 10.27% of the controls had an AL 6 or above. When compared between cases and controls, a significant difference in the distribution of AL scores was observed between the prostate cancer cases and controls (P < 0.001). Compared to the controls, prostate cancer cases were less likely to have AL scores of 0 to 2 and more likely to have AL scores of 3 and above. As a continuous variable, the mean AL of the prostate cancer cases was 3.47, statistically significantly higher than the 3.35 of the controls (P < 0.001).

**Table 2:** AL scores and score categories between prostate cancer cases and controls.

	Cancer-free controls (N = 154,673)	Incident cases (N = 7,291)	P value
AL (continuous), mean (SD)	3.35 (1.67)	3.47 (1.58)	< 0.001
AL category, N (%)			
0	5719 (3.70)	161 (2.21)	< 0.001
1	16,536 (10.69)	644 (8.83)	
2	26,736 (17.29)	1,253 (17.19)	
3	34,367 (22.22)	1,662 (22.80)	
4	32,064 (20.73)	1,658 (22.74)	
5	23,362 (15.10)	1,167 (16.01)	
6 and over	15,889 (10.27)	746 (10.23)	

Then, we compared the AL score between prostate cancer cases and controls by selected characteristics **(**Table 3**).** Compared to the cancer-free controls, the mean AL for each selected characteristic was statistically significantly higher in the cases (P < 0.05). However, among older men (≥ 58 years old) and retired men, the mean AL was lower in cases than in controls (P < 0.001 and P = 0.004, respectively). Then, we compared the AL score by selected characteristics within the cases and controls separately (Table 3). Among the cancer-free population, compared to their counterparts, older men (≥ 58 years old), less educated, unemployed, retired, ever smokers, heavy drinkers, and having a lower income (≤ £30,999), no family history of cancer, moderate/high total physical activity and sometime/frequent sleeplessness had statistically significant higher AL score (P < 0.05). A similar trend was also observed among cases, except for total physical activity and alcohol consumption. Additionally, in both cases and controls, the mean AL score differed among racial groups (P < 0.01).

**Table 3:** Comparison of AL score by selected characteristics between prostate cancer cases and non-cancer controls.

	Cancer-free controls (N = 154,673)	Incident cases (N = 7,291)	P value
	AL, mean (SD)	AL, mean (SD)	
Age at recruitment, median			
< 58 years old	3.08 (1.72)	3.16 (1.63)	0.036
≥ 58 years old	3.64 (1.58)	3.56 (1.55)	< 0.001
P value	< 0.001	< 0.001	
Race			
White	3.36 (1.67)	3.47 (1.58)	< 0.001
Black	2.86 (1.77)	3.45 (1.65)	< 0.001
Asian	3.55 (1.66)	3.39 (1.42)	0.284
Mixed or others	3.20 (1.74)	3.58 (1.67)	0.067
P value	< 0.001	< 0.001	
Family history of cancer			
Yes	3.25 (1.66)	3.34 (1.61)	0.139
No	3.41 (1.67)	3.48 (1.56)	< 0.001
P value	< 0.001	0.042	
Education			
High school or less	3.40 (1.66)	3.54 (1.53)	< 0.001
College / professional	3.10 (1.67)	3.24 (1.60)	< 0.001
P value	< 0.001	< 0.001	
Employment			
Unemployment	3.74 (1.76)	3.86 (1.69)	0.193
Employment	3.17 (1.68)	3.33 (1.60)	< 0.001
Retired	3.66 (1.57)	3.58 (1.53)	0.004
P value	< 0.001	< 0.001	
Income			
< £30,999	3.58 (1.66)	3.63 (1.59)	0.089
≥ £30,999	3.14 (1.65)	3.30 (1.55)	< 0.001
P value	< 0.001	< 0.001	
Townsend index			
Low	3.31 (1.64)	3.43 (1.56)	< 0.001
High	3.40 (1.70)	3.51 (1.50)	< 0.001
P value	< 0.001	0.028	
Cigarette smoking			
Never	3.15 (1.67)	3.31 (1.58)	< 0.001
Ever	3.55 (1.65)	3.62 (1.57)	0.008
P value	< 0.001	< 0.001	
Total physical activity			
Low	3.51 (1.77)	3.57 (1.55)	0.341
Moderate/High	3.33 (1.66)	3.45 (1.56)	< 0.001
P value	< 0.001	0.071	
Alcohol consumption			
Never/light/moderate	3.39 (1.73)	3.50 (1.65)	< 0.001
Heavy	3.32 (1.62)	3.44 (1.53)	< 0.001
P value	< 0.001	0.139	
Sleeplessness			
Never/rarely	3.23 (1.66)	3.40 (1.54)	< 0.001
Sometimes/frequent	3.41 (1.67)	3.50 (1.60)	< 0.001
P value	< 0.001	0.016	

**Fig. 1:**
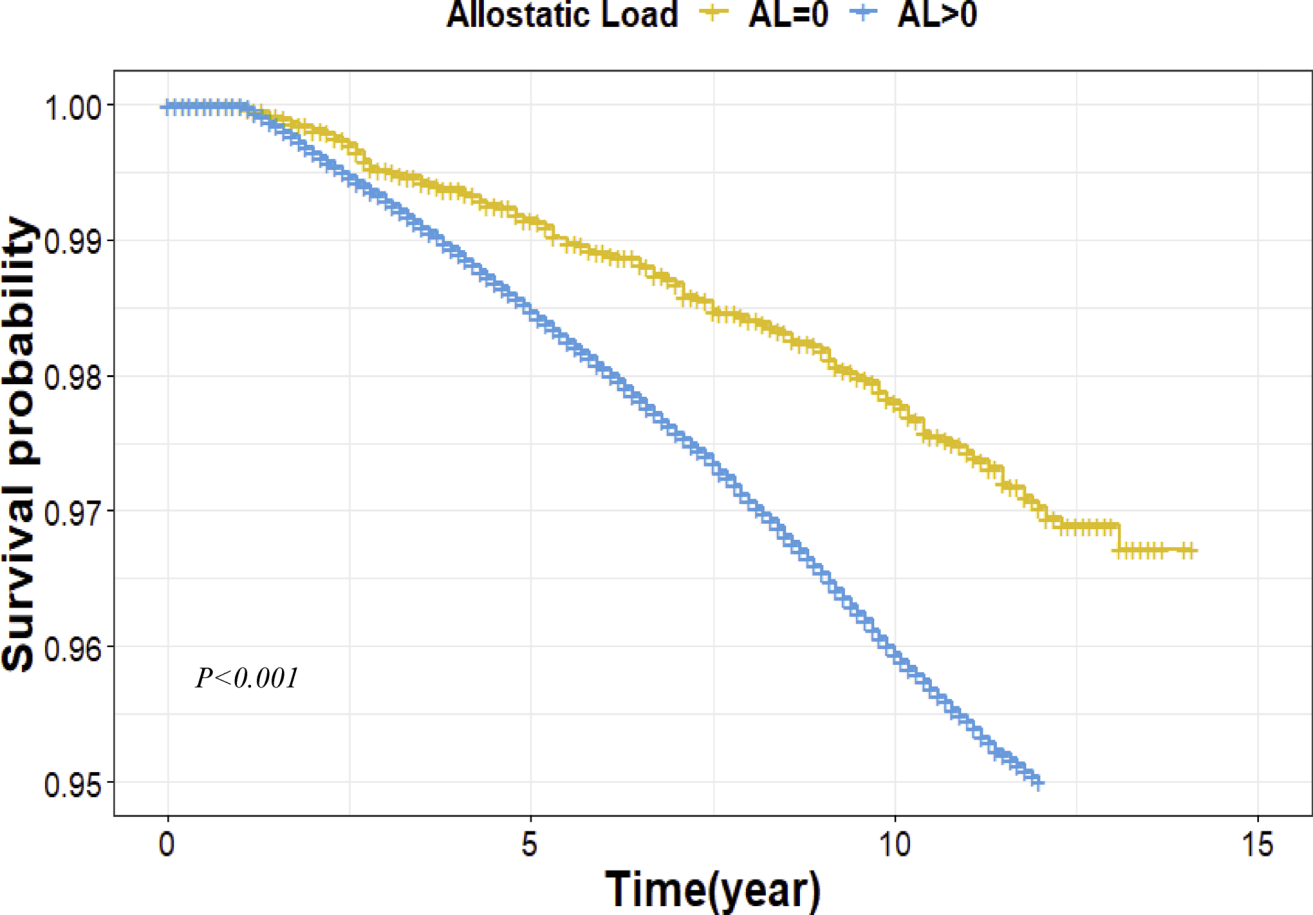
Kaplan Meier analysis to assess the relationship between AL and prostate cancer risk.

Next, we investigated the association between AL and prostate cancer risk (Table 4). Firstly, we treated AL as a continuous variable. In the univariate Cox Proportional Hazard regression model, we observed a 5% increased risk of prostate cancer per one AL unit increase (HR = 1.05, 95%CI 1.03, 1.06). The Kaplan–Meier survival curves for the association between the AL score and prostate cancer risk are shown in Fig. 1. However, the association was no longer significant in the multivariate Cox regression analysis after adjusting age, race, and family history of prostate cancer, education, cigarette smoking, total physical activity, alcohol consumption, sleep quality, and PRS (HR = 1.00, 95%CI 0.99, 1.02). As presented above, the mean AL was higher in prostate cancer cases and controls among younger men (< 58 years old) (P = 0.036) but lower in prostate cancer cases and controls among older men (≥ 58 years old) (P < 0.001). To dissect the impact of age on the relationship between AL and prostate cancer risk, we further stratified the study population into two groups (< 58 and ≥ 58 years old). Among younger men, in the multivariate Cox regression analysis, we observed a 4% increased risk of prostate cancer per one AL unit increase (HR = 1.04, 95%CI 1.01, 1.07). Then, we treated AL as a categorical variable. Among younger men, compared to those with AL of 0, those with ALs of 1, 2, 3, 4, 5, and ≥ 6 had a statistically significantly increased risk of prostate cancer with HRs of 1.52, 1.63, 1.60, 1.84, 1.72, and 1.59, respectively. A statistically significant increasing trend of prostate cancer risk was also observed (p for trend = 0.003). On the other hand, among older men, no statistically significant association was observed between AL as a continuous variable or a categorical variable and prostate cancer risk.

**Table 4:** Associations between AL scores and AL categories with prostate cancer risk.

	Univariate, HR (95% CI)	P value	Multivariate* HR (95% CI)	P value
AL, continuous, Per one unit	1.05 (1.03, 1.06)	< 0.001	1.00 (0.99, 1.02)	0.910
Stratified by age				
	< 58 years old*	≥ 58 years old*		
AL, continuous, Per one unit	1.04 (1.01, 1.07)	< 0.001	0.99 (0.97, 1.00)	0.910
AL (categorical)				
0	Reference		Reference	
1	1.52 (1.16, 2.01)	0.003	0.96 (0.77, 1.20)	0.720
2	1.63 (1.25, 2.13)	< 0.001 s	1.11 (0.83, 1.25)	0.872
3	1.60 (1.23, 2.09)	0.001	1,00 (0.81, 1.23)	0.977
4	1.84 (1.41, 2.41)	< 0.001	0.99 (0.81, 1.22)	0.953
5	1.72 (1.30, 2.27)	< 0.001	0.96 (0.78, 1.19)	0.728
6 and over	1.59 (1.18, 2.14)	0.002	0.92 (0.74, 1.14)	0.464
P for trend	0.003		0.167	

*Adjusted by gender, race/ethnicity, education, family history of cancer, cigarette smoking, alcohol consumption, physical activity, sleep quality, and PRS.

**Fig. 2:**
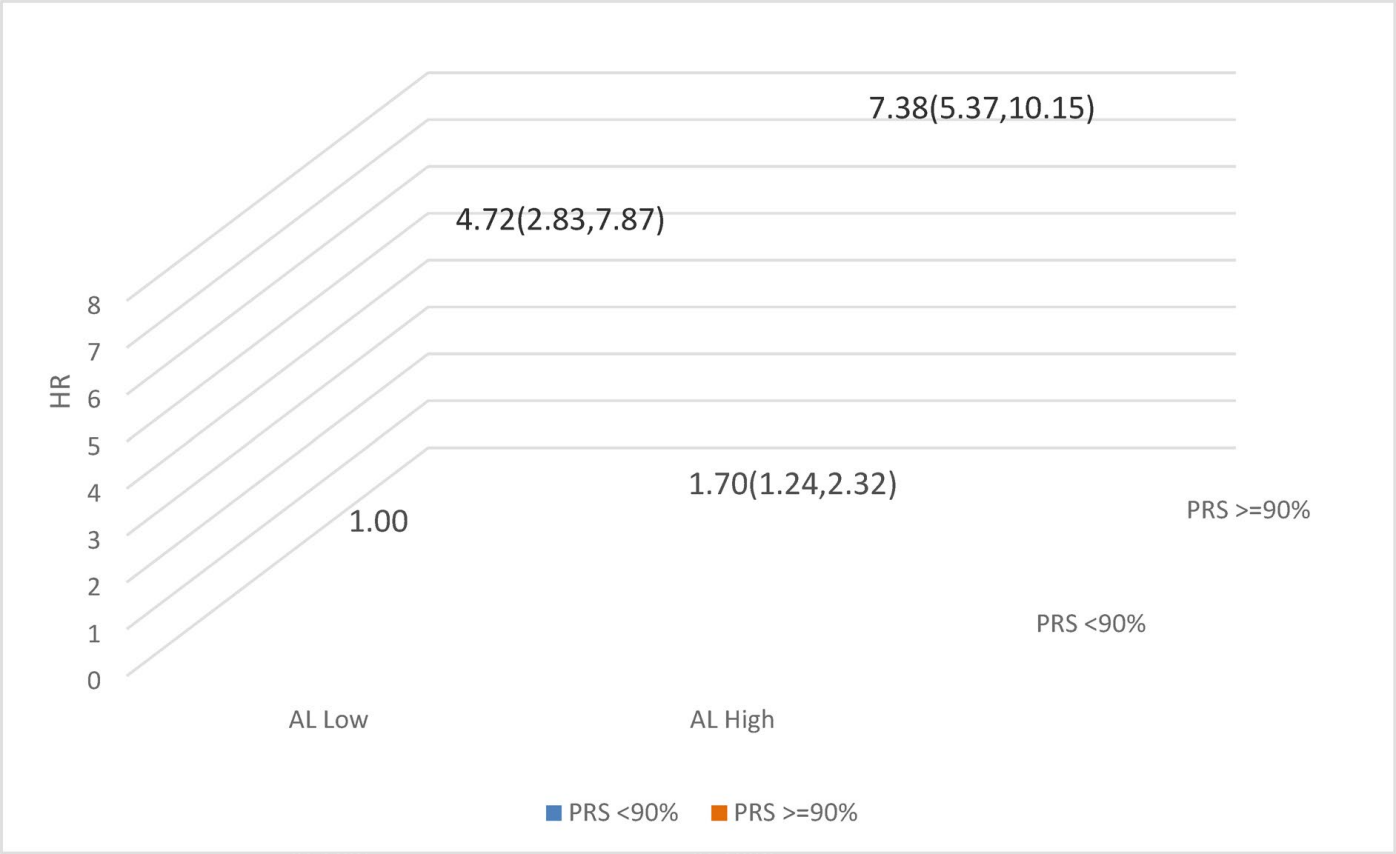
Joint effect between AL and prostate cancer risk among younger men.

Finally, we assessed whether there was a joint effect between AL and PRS of prostate cancer among younger men (Fig. 2). A more than an additive joint effect was observed. Compared to those with lower AL (AL = 0) and PRS < 90%, those with higher AL (AL > 0) and PRS ≤ 90% and those with lower AL and PRS ≥ 90% had 1.70 and 4.72-folds increased risk of prostate cancer (HR = 1.70, 95% CI 1.24, 2.32; HR = 4.72, 95% CI 2.83, 7.87), and those with both high AL and PRS ≥ 90% had 5.63-fold increased risk of prostate cancer (HR = 7.38, 95% CI 5.37, 10.15).

## Discussion

To our knowledge, this is the first study to assess the relationship between AL and the development of prostate cancer in a large cohort study. We found levels of AL score were higher in prostate cancer cases than in non-cancer controls (3.47 vs 3.35, P < 0.001). In the risk assessment, a significant association between AL score and prostate cancer risk was observed among men younger than 58 years old. Among them, we observed a 4% increased risk of prostate cancer per one AL unit increase (HR = 1.04, 95%CI 1.01, 1.07). A significant association was confirmed if AL was treated as a categorical variable. However, no significant association was observed among older men (≥ 58 years old). In addition, a more than multiplicative and additive joint effect between AL and PRS of prostate cancer was observed among younger men. So far, there is only one meeting abstract from a small cross-sectional study to report that higher AL is associated with more aggressive phenotypes of prostate cancer, including de novo metastatic disease, earlier progression to castrate-resistance, and higher Gleason scores^13^. Due to the lack of tumor characteristics and clinical information in the UK Biobank, we can’t assess the relationship between AL and prostate tumor characteristics in our study. However, the results from both studies point out that AL may play an important role in prostate carcinogenesis.

Given AL is a biomarker of chronic stress, our finding is consistent with literature reports that chronic stress may contribute to prostate carcinogenesis. Hassan et al. reported that behavioral stress could inhibit apoptosis and promote prostate cancer development in mice^5^. In adult male rats, repeated stress could significantly increase the transcript levels of several genes associated with cellular proliferation, including proto-oncogenes in prostate tissues^4^. In addition, Bellinger et al. found that psychological stress could drive tumor inflammatory cytokines and accelerate prostate cancer growth in mice^20^. In population-based studies, a significant association between stress and prostate cancer risk is also reported^6,21–23^. In a case–control study from Canada, perceived workplace stress was found to be associated with an increased risk of prostate cancer before age 65^21^. This age difference is consistent with our findings that the risk association between AL and prostate cancer risk was only exit among younger men (< 58 years old). In another study to assess the relationship between distress and prostate cancer diagnosis, men with ‘possible’ clinical depression at initial PSA testing were found to be 23% more likely to have a diagnosis of prostate cancer^22^. Similar results were also reported in a case–control study among older African American and Caucasian men (aged 65 to 79 years old) from South Carolina^23^. In a cohort of World Trade Center responders (N = 6857), re-experiencing the stressful events of 9/11/2001 was found to be associated with increased prostate cancer incidence (HR = 1.96, 95%CI 1.26, 3.05), even upon adjusting for confounders^6^. Meanwhile, in an early study from the Copenhagen City Heart Study, no significant association was observed between stress and prostate cancer risk (HR = 0.99, 95%CI 0.90, 1.09)^7^. Thus, results from cell lines, animal models, and human studies have supported the relationship between AL and prostate cancer risk reported in this study.

It has been well-known the incidence of prostate cancer is higher among African American men than their White counterparts. Interestingly, in early 2000, Dr. Gary Ellison and his colleagues proposed a theoretical framework for an association between psychological stress and prostate cancer^24^. The framework suggests that socioeconomic disadvantage is more prevalent among African Americans than among European American men, which may consequently lead to higher levels of psychological stress among African American men. African American men also routinely experience racism-induced stress^25^. Coupling them together, within the context of history and culture, elevated psychological stress may partially explain the higher incidence of prostate cancer among African Americans. Unfortunately, we cannot assess such a framework in this study. However, we found that AL is affected by SES-related variables, including education, employment, income, and the Townsend Deprivation Index. Specifically, among both non-cancer controls and prostate cancer cases, we found that men with lower levels of education, unemployment, lower income, and higher levels of Townsend Deprivation Index had statistically significantly higher levels of AL score (P < 0.05, respectively). Our data highlights the close relationship between SES at both individual and neighborhood levels with chronic stress and AL.

However, among non-cancer controls, we observed that the mean AL was lower among Black participants compared to White participants (Table 3), and this difference persisted after adjustment for covariates. This finding is not consistent with much of the existing literature^16,17,26–28^. Several factors may contribute to the discrepancy. First, this pattern may reflect selection bias or the well-documented healthier volunteer effect in the UK Biobank^29^. Individuals who participate in large cohort studies tend to be healthier and more socioeconomically advantaged than the general population, and this bias may be more pronounced among racial and ethnic minority groups. Consequently, Black participants included in the control sample may represent a particularly healthy subgroup with lower AL than would be expected in the general Black population. Second, the number of Black participants in the control group is considerably smaller than that of White participants, which may reduce the precision and generalizability of the group-level AL estimates. Finally, survivor bias may also play a role, as individuals with higher AL are more likely to experience earlier onset of chronic disease or premature mortality, potentially making them less likely to be recruited into the cohort.

One surprising observation in this study is that the mean AL was higher in prostate cancer cases and non-cancer controls among younger men (< 58 years old) (P = 0.036) but lower in prostate cancer cases than controls among older men (≥ 58 years old) (P < 0.001). The age difference is possibly due to other covariates that may confound the association. In further analysis, we adjusted a series of covariates in the analysis (e.g., race, education, family history of cancer, cigarette smoking, alcohol consumption, total physical activity, and sleep quality), the significant association among older men was no longer existed (P = 0.069). In further risk assessment, the significant association between AL and prostate cancer risk is only evident among younger men (< 58 years old). A similar observation was also reported by lanc-Lapierre et al. that perceived workplace stress was found to be associated with an increased risk of prostate cancer before age 65^21^. We don’t have an explanation for the observed age difference. However, it may fit well with the Strength and Vulnerability Integration SAVI model^30^ which suggests that older adults often develop strengths through a lifetime of experiences and are usually better able to negotiate through challenges better than younger adults. In the case of AL and prostate cancer, older men may develop better-coping skills with chronic stress and get more resilience over time. Thus, the impact of AL on prostate cancer may be less hostile among them than younger men. It might also reflect a cohort effect, whereas stressors would differ depending on the era in which they occurred. More studies are needed to clarify the association further.

Our previous work using this same cohort found that higher AL was associated with an increased risk of breast cancer and the association was more evident among older women (≥ 57 years old)^15^. Combining finding from those two studies, it suggests that the association between AL and cancer risk may vary by gender and age. It may reflect underlying variations in stress biology, hormonal regulation, and immune function across the life course and between sexes^10^. For instance, AL has been more strongly associated with breast cancer incidence among older women, potentially due to postmenopausal changes in estrogen levels, which influence inflammatory and immune pathways involved in tumor development^31^. In contrast, elevated AL appears to be associated with prostate cancer risk primarily in younger men, when androgen levels and stress reactivity are highest^32^. Gender differences in stress response systems, such as greater hypothalamic–pituitary–adrenal (HPA) axis activation and glucocorticoid sensitivity in women, and more pronounced sympathetic nervous system (SNS) responses in men, may also modulate the physiological consequences of chronic stress differently^33–35^. Additionally, behavioral and psychosocial coping mechanisms, which often differ by gender and age, may contribute to variation in AL and its health effects. These findings highlight the importance of considering both sex and age when evaluating the biological embedding of chronic stress in cancer etiology and outcomes.

One interesting finding from this study is the more than additive joint effect between AL and PRS of prostate cancer among younger men. Compared to the reference group (AL = 0 and PRS < 90%), groups with AL > 0 and PRS < 90%, AL = 0 and PRS ≥ 90%, and AL > 0 PRS ≥ 90% had 1.70, 4.72, and 7.38-folds increased risk of prostate cancer. If further confirmed in other studies, it raises the possibility of using AL in prostate cancer risk stratification and even possibly risk prediction. Due to the lack of serum prostate-specific antigen (PSA) screening information in the UK Biobank, we can’t assess the role of AL in PSA screening. The measurement of serum PSA plays an essential role in the early detection of prostate cancer. Concerns are raised by the poor specificity of the PSA test and the lack of conclusive evidence that early detection and treatment of prostate cancer carries survival benefits. Previous studies have shown PSA levels were not affected by psychological stress^22^. So, it would be interesting to assess whether AL may play any role in PSA screening.

There were two significant limitations in this study. First, given that about 95% of the study population is White, this study lacks minorities. This may limit the generalizability of the findings in more diverse populations. Notably, Black men are known to have a higher incidence and mortality rate of prostate cancer. In addition, they are known to have higher levels of chronic stress than their white counterparts, so research on Black men is significant and needed. Second, the UK Biobank lacks information on tumor characteristics and detailed clinical information. So, we cannot assess the role of AL in prostate tumor aggressiveness, particularly clinically significant prostate cancer. In addition, we acknowledge the potential impact of missing data on the construction of the allostatic load (AL) score. To assess this, we compared participants with complete AL data (n = 161,964) to those with one or more missing AL components (n = 50,549). A chi-square test indicated no significant difference in prostate cancer incidence between the two groups (p = 0.33), suggesting that exclusion due to missing AL data is unlikely to have biased the observed association with prostate cancer risk.

In summary, we carried out the first study to assess the role of AL on the risk of prostate cancer in a large cohort. We found that higher AL was associated with an elevated risk of prostate cancer in younger men (< 58 years old). In addition, a more than multiplicative and additive joint effect between AL and PRS of prostate cancer among younger men was observed. Thus, AL, as a biomarker of chronic stress, could be useful in prostate cancer risk stratification and prediction. Other cohort studies must further confirm the results, particularly those with a large number of minorities.

## Supplementary Information

Below is the link to the electronic supplementary material.

Supplementary Material 1.

## Data Availability

All data generated or analyzed during this study are included in this published article and its supplementary information files.
