# Supplementary material for: Allostatic load and risk of prostate cancer in UK Biobank
Source: Sci Rep. 2025 Oct 27;15:37505. doi: 10.1038/s41598-025-21510-8 (PMC12559758; doi:10.1038/s41598-025-21510-8)
Supplement: Supplementary file 1 — Supplementary Material 1. [file 41598_2025_21510_MOESM1_ESM.docx]

Supplements:

Table1: Codes used to identify prostate cancer cases (Study censoring date:12/31/2020)

| **Categories** | **Frequency (%)** | **ICD10 codes** |
| --- | --- | --- |
| **Prostate cancer cases** | | |
| - Prevalent: | 3042 (1.33%) | Codes start with C61 |
| - Incident: | 10190(4.45%) [9514(Incident >1 year);  676(Incident <1 year))] |  |
| **Other cancers(except for non-melanoma skin cancer )** | | |
| - Prevalent: | 14794(6.46%) | Codes start with C except codes for PC and non-melanoma skin cancer |
| - Incident: | 5439(2.38%) |  |
| **In situ carcinoma** | | |
| - Prevalent: | 2277(0.99%) | Codes start with D0-D09 |
| - Incident: | 883(0.39%) |  |
| **Neoplasm of unknown nature or behavior** | | |
| - Prevalent: | 1030(0.45%) | Codes start with D37- D48 |
| - Incident: | 646(0.28%) |  |
| **Benign neoplasms** | | |
| - Prevalent: | 254(0.11%) | Codes start with D10-D36 |
| - Incident: | 234(0.10%) |  |
| **non-melanoma skin cancer** | | |
| - Prevalent: | 12198(5.33%) | Codes start with C44 |
| - Incident: | 43591.90%) |  |
| **Unknow prevalent cancers** | 1218(0.53%) | Subject only with cancer diagnosed time, no Codes |
| **Non-case controls** | 172446 (75.30%) | Remaining codes or subjects with no code assigned |
| **Total** | 229010 (100%) | |

- Subject with any cancer developed before enrolment was excluded.
- Subject with any cancer other than prostate cancer during follow-up were censored at the date of diagnosis.

Table2: Distribution and high-risk cutoff points for individual biomarkers of AL scores (N=212409)

|  | Cutoff Value | Mean(SD) | N (%) at Risk | N (%)missing |
| --- | --- | --- | --- | --- |
| Waist to hip ratio | >=0.9 for male | 0.94(0.07) | 150440(70.83%) | 1056(0.50%) |
| Pulse rate | >100 | 68.28(11.87) | 2472(1.16%) | 12625(5.94%) |
| SBP (mm Hg) | >=140 | 140.70(17.43) | 96772(45.56%) | 12627(5.94%) |
| DBP (mm Hg) | >=90 | 84.08(10.03) | 55045(25.91%) | 12625(5.94%) |
| HDL (mmol/L) | >1 for male | 1.28(0.31) | 31995(15.06%) | 28443(13.39%) |
| LDL ( mmol/L) | >3.4 | 3.48(0.86) | 105135(49.50%) | 13557(6.38%) |
| Total Cholesterol(mmol/L) | >5.2 | 5.49(1.13) | 118263(55.68%) | 13119(6.18%) |
| Triglycerides(mmol/L) | >=1.7 | 1.98(1.16) | 98938(46.58%) | 13336(6.28%) |
| C-reactive protein( mg/L) | >3 | 2.42(4.25) | 39671(18.68%) | 13644(6.42%) |
| Creatinine(umol/L) | >114.9 for male | 81.54(18.60) | 3756(1.77%) | 13230(6.23%) |
| Glycated hemoglobin (HbA1c)mmol/mol | >48 | 36.49(7.63) | 9934(4.68%) | 14429(6.79%) |
| Medication history | Yes |  | 68294(32.15%) | 4313(2.03%) |

| Table 3. Association between age and AL factors in cases and controls | | | |
| --- | --- | --- | --- |
|  | Case-Coefficient,95%CI | Control- Coefficient,95%CI | Pvalue |
| SBP (mmHg) | 0.546 (0.478, 0.614) | 0.561 (0.551, 0.571) | 0.67 |
| DBP (mmHg) | -0.090 (-0.129, -0.052) | -0.001 (-0.008, 0.005) | <0.01 |
| PR (bpm) | -0.010 (-0.055, 0.036) | -0.012 (-0.019, -0.004) | 0.94 |
| CRP (mg/L) | 0.029 (0.014, 0.044) | 0.030 (0.028, 0.033) | 0.88 |
| Creatinine (µmol/L) | 0.158 (0.103, 0.214) | 0.152 (0.141, 0.164) | 0.87 |
| HDL (mmol/L) | 0.001 (-0.000, 0.002) | 0.002 (0.001, 0.002) | 0.45 |
| LDL (mmol/L) | -0.022 (-0.025, -0.018) | -0.015 (-0.015, -0.014) | <0.01 |
| Total Cholesterol (mmol/L) | -0.027 (-0.031, -0.022) | -0.017 (-0.018, -0.016) | <0.01 |
| TG (mmol/L) | -0.009 (-0.013, -0.005) | -0.006 (-0.007, -0.005) | 0.12 |
| HbA1c (mmol/mol) | 0.137 (0.111, 0.163) | 0.145 (0.141, 0.150) | 0.58 |
| WHR | 0.001 (0.001, 0.001) | 0.002 (0.002, 0.002) | 0.03 |
